# Optimizing Greenhouse Design with Miniature Models and IoT (Internet of Things) Technology—A Real-Time Monitoring Approach

**DOI:** 10.3390/s24072261

**Published:** 2024-04-02

**Authors:** Ioana Udrea, Viorel Ionut Gheorghe, Angel Madalin Dogeanu

**Affiliations:** 1 Department of Mechatronics and Precision Mechanics, Faculty of Mechanical Engineering and Mechatronics, National University of Science and Technology Politehnica Bucharest, 060042 Bucharest, Romania; viorel.gheorghe@upb.ro; 2 Faculty of Building Services, Technical University of Civil Engineering Bucharest, 020396 Bucharest, Romania

**Keywords:** Internet of Things, heat transfer, energy balance, ThingSpeak platform, temperature sensor, humidity sensor, mathematical model

## Abstract

The market for smart greenhouses has been valued at USD 1.77 billion in 2022 and is expected to grow to 3.39 billion by 2030. In order to make this more efficient, with the help of Internet of Things (IoT) technology, it is desired to eliminate the problem of traditional agriculture, which has poor monitoring and accuracy control of the parameters of a culture. Climate control decisions in a greenhouse are made based on parameter monitoring systems, which can be remotely controlled. Instead of this adjustment of the measured parameters, it would be preferable from the point of view of energy consumption that they should be calculated at optimal values from the design phase of the greenhouse. For this reason, it would be better to perform an energy simulation of the greenhouse first. For the study carried out in this work, a small greenhouse (mini-greenhouse) was built. It was equipped with an IoT sensor system, which measured indoor climate parameters and could send data to the cloud for future recording and processing. A simplified mathematical model of the heat balance was established, and the measured internal parameters of the mini-greenhouse were compared with those obtained from the simulation. After validating the mathematical model of the mini-greenhouse, this paper aimed to find the optimal position for placing a normal-sized greenhouse. For this, several possible locations and orientations of the greenhouse were compared by running the mathematical model, with which the most unfavorable positions could be eliminated. Then, some considerably cheaper “mini-greenhouses” were made and placed in the locations with the desired orientations. Using sensor systems and technologies similar to those presented in this work, the parameters from all mini-greenhouses can be monitored in real time. This real-time monitoring allows for the simultaneous analysis of all greenhouses, without the disadvantages of data collection directly in the field, with all data being recorded in the cloud and other IoT-specific advantages being made use of. In the end, we can choose the optimal solution for the location of a real-size greenhouse.

## 1. Introduction

The Food and Agriculture Organization (FAO) believes that food production will need to increase by 70% to feed an estimated global population of 9 billion in 2050 [1]. In this context, the market for smart greenhouses is growing rapidly [2].

Agriculture based on the use of greenhouses is very important in the agricultural sector. It helps to cover the demand for food worldwide by ensuring a suitable microclimate for plants, which leads to increased productivity and better-quality yields [3].

Many models have been used over time to calculate the heat transfer of a greenhouse. Static models [4,5] have lower precision, but are easy to control, while dynamic models have higher precision, though it is more difficult to find calculation tools for them—withthese being usually specific to buildings [6,7,8,9]. TRNSYS, EnergyPlus, and other dynamic simulation software have also been used to model greenhouses. Thus, Vadiee [10] looked for the elements with the greatest impact on the energy balance of the greenhouse. He studied the concept of the “closed greenhouse” and the potential for storing heat in such a greenhouse. An important element of impact in terms of required heating and cooling loads is greenhouse orientation [11]. In addition, the possibility of using thermal screens for energy-saving purposes must be considered. In this sense, an extensive study was carried out with the help of a simulation model in TRNSYS [12]. In most of the available models, the air density is considered constant [13]. The effect of air density variations on the greenhouse temperature model is important, and there [13,14] are studies in this regard. There is also the neural-network-based approach to modeling the climate in a greenhouse, such as that shown in the study of Ferreira [15].

Another important aspect of a greenhouse is the evapotranspiration of plants, which is the loss of plant water in the processes of transpiration and evaporation. There are greenhouses where crops are grown in soil and greenhouses where they are grown in substrates [16]. Plant evapotranspiration depends on the level of irrigation. Studies on the irrigation management of European greenhouse vegetable crops were carried out by Incrocci [17]. Greenhouses can also be used as a drying system, in which the behavior of water vapor is very important [18].

As shown in [19,20,21,22], convective heat transfer coefficients between the inner cover surface and the air depend on the temperature difference between the inside and outside air. The convective heat transfer coefficient depends, of course, on the external wind speed, for which there are empirical calculation formulas. Another aspect that must be considered when discussing convective transfer is vertical temperature variation in a greenhouse. Complex calculation models have been developed to determine this thermal stratification [23,24,25].

Recent research focuses on increasing greenhouse thermal efficiency and maximizing solar energy use. For this, the active solar water curtain heating system [26] was conceived. Recent studies also focus on reducing greenhouse consumption [27,28], and this is best carried out in the design stage rather than when the greenhouse has already been built. The assessment of potential renewable energy alternatives, with emphasis on the photovoltaics energy for a greenhouse, also represents a current trend [29,30,31]. For greenhouses to be more efficient, the use of renewable energy sources must be correlated with the reduction in energy required for cooling and heating.

This study aims to find a solution for determining the optimal location of a greenhouse, proposing a combined method composed of simulation and preliminary experimental tests. Various orientations and locations of a real-size greenhouse will be analyzed for this purpose. Some of them will be eliminated after running a mathematical model that will calculate the temperature in the greenhouse. After this, small greenhouses, incurring low costs, will be positioned in the remaining locations in order to take measurements in them. All of the measured data will be centralized in a ThingSpeak [32] dashboard. The right decisions will be made faster than if the data are collected in the field. In support of this method, the mathematical model must first be developed, which will be made for a mini-greenhouse. Next, it will be validated by comparing the results obtained from the measurements in a mini-greenhouse with those obtained from an energy balance in a steady-state regime. Some approximations are made in this assimilation of a small greenhouse with a large one, in terms of vertical air stratification; however, the purpose of this work is not to determine, as precisely as possible, the parameters inside of the greenhouse, but to determine the comparative best solution among those that will be taken into account. The low costs for the realization of a small greenhouse allow the later realization of other geometric models of greenhouses and the verification of the mathematical model as well. At the same time, the paper also proposes a solution for sending the mini-greenhouse data to the cloud. The measurement system will be an intelligent one that can transmit the read data into the cloud, thus taking advantage of the cloud’s data security, as they can easily be stored in a database [33]; moreover, in terms of scalability, several similar systems can be tracked centrally from the same application and others.

## 2. Materials and Methods

In this section, the built small greenhouse is presented, which can be very easily used. Also, the sensor system used to make the measurements and the equations used for modeling the greenhouse heat transfer are presented.

### 2.1. Mini-Greenhouse Description

**Figure 1:**
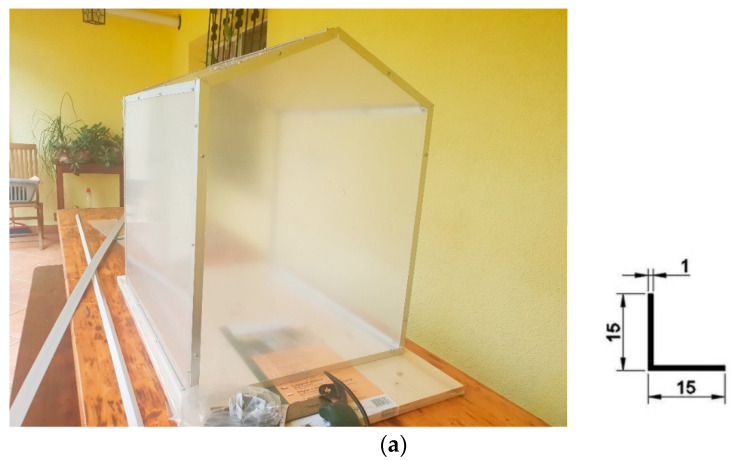

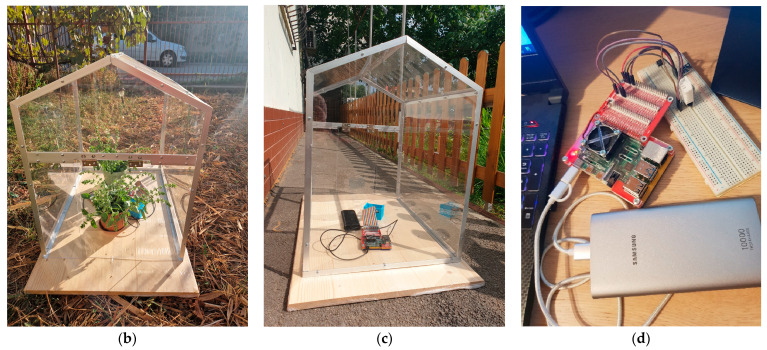
Stages in the life cycle of the mini-greenhouse. (**a**) The mini-greenhouse: under construction; (**b**) The mini-greenhouse: outdoors, with the sensors working in 2022; (**c**) The mini-greenhouse: outdoors, with the sensors working in 2023; (**d**) The sensor system used in this study, powered by an external charger.

The mini-greenhouse, with the sensor system working inside of it, can be seen in Figure 1b. The sensor system is powered by an external battery and connected to the Wi-Fi network of the building next to which the mini-greenhouse is situated. This is an advantage of the small greenhouse, as it can be placed anywhere.

The mini-greenhouse is made of plexiglass, which is mounted on aluminum profiles with screws. The mini-greenhouse can be seen in the construction stage, as well as the dimensions of the aluminum corner profile, in Figure 1a. The mini-greenhouse can be seen “at work”, with the sensor system inside, in Figure 1b,c. The measurements were made in the summer of 2022 (Figure 1b) and in the autumn of 2023 (Figure 1c). The positioning of the mini-greenhouse in different locations can also be seen in the two figures. For the tests performed for this paper, the mini-greenhouse was placed, as can be seen, on a wooden support. The ground area of the greenhouse is 450 mm × 600 mm, and height is 600 mm.

### 2.2. Sensor System

A measuring system consisting of two sensors, a DHT22 (temperature and humidity sensor) [34] and a BMP180 (temperature and pressure sensor) [35], is placed inside of the greenhouse. The sensors are mounted on a RaspberryPi Board [36], using a multiplexing board for GPIO port expansion (see Figure 1d).

The entire system is presented in [37]. A software, written in Python, runs on the RaspberryPi operating system, which transmits the read data to the cloud in the ThingSpeak platform [32]. At the same time, the values are sent to the terminal on the RaspberryPi system. The free version of ThingSpeak limits channel updates to 15 s. The code on the RaspberryPI system sends data to the ThingSpeak platform every 15 s.

ThingSpeak provides data in the form of graphs or numerical values. The numerical data provided by ThingSpeak are in JSON format. In this file, the recorded values have a well-established hierarchical structure, specific to this type of file. They can be read through the HTTP GET method, made through a query in URL format with the syntax of https://api.thingspeak.com/channels/<channel_id>/feeds.<format>. The channel for which the reading is desired must be specified, as well as other parameters representing the format in which the data will be presented (HTTP response). For example, entries in JSON format, hours of entries in JSON format, and others. In the case of this work, the request “https://api.thingspeak.com/channels/946567/feeds.json?results=50” (accessed on 30 November 2023) is sufficient.

ThingSpeak has certain limitations in terms of being customized exactly according to the application’s requirements. This is especially true if several similar systems are used, with each being located in a distinct mini-greenhouse, where the centralization of data from all of these systems will be necessary. For this, the application that reads the data from the sensors, now made in Python (Version 3.11.0, 2022), can be rewritten, and, with the help of web frameworks like ReactJS (Version 18.1.0, 2022) or Angular (Version 15.0.0, 2022), all of the customizations considered to be useful can be obtained.

### 2.3. The Principles of Modeling the Greenhouse Heat Transfer

The model built for determining the indoor temperature of the mini-greenhouse uses the equation of energy balance in a steady state. The law of energy conservation is applied and the terms containing the indoor temperature are identified. In this calculation, the stored energy is neglected, because the internal heat capacity of the plants and air is very low [38]. The air in the greenhouse is considered to be well mixed, which implies a lack of spatial variation of temperature.

The energy balance equation is as follows:

(1) $$Q_{solar}-Q_{e}-Q_{cond}-Q_{v}-Q_{t}=0$$

where:

Q_solar_ [W]—total solar radiation;
Q_e_ [W]—latent heat energy flux due to evapotranspiration of the plant;
Q_cond_ [W]—heat energy flux transferred by conduction and convection;
Q_v_ [W]—heat energy flux transferred due to air exchange;
Q_t_ [W]—heat energy flux lost by the transfer of longwave radiation.

The energy received from the sun, Q_solar_, is calculated with the following formula:

(2) $$Q_{solar}=\sum \tau \cdot S_{hading}\cdot G_{T}\cdot A_{si}$$

(3) $$G=G_{d}+G_{b}$$

(4) $$G_{T}=G_{bT}+G_{dT}+G_{rT}$$

(5) $$G_{bT}=G_{b}\cdot \cos i_{s}$$

(6) $$G_{dT}=G_{d}\cdot \frac{\left(1+\cos\beta \right)}{2}$$

(7) $$G_{rT}=G\cdot \rho \cdot \frac{\left(1-\cos\beta \right)}{2}$$

(8) $$\cos i_{s}=\cos\beta \cdot \sin\alpha_{s}+\sin\beta \cdot \cos\alpha_{s}\cdot \cos\left(\gamma -\gamma_{s}\right)$$

where:

τ—transmissivity of the mini-greenhouse material;
S_hading_—shading coefficient;
G [W/m^2^]—global irradiance on the horizontal plane received per unit area and per unit time;
G_b_ [W/m^2^]—beam horizontal irradiance;
G_d_ [W/m^2^]—diffuse horizontal irradiance;
G_T_ [W/m^2^]—irradiance on tilted surfaces;
G_bT_ [W/m^2^]—beam irradiance on tilted surfaces;
G_dT_ [W/m^2^]—diffuse irradiance on tilted surfaces;
G_rT_ [W/m^2^]—reflected irradiance;
A_si_ [m^2^]—the receiving surface area;
i_s_—incidence angle of the sun rays;
β—surface’s inclination angle with regard to the horizontal plane;
γ—surface’s azimuth;
γ_s_—solar azimuth;
α_s_—solar altitude.

**Figure 2:**
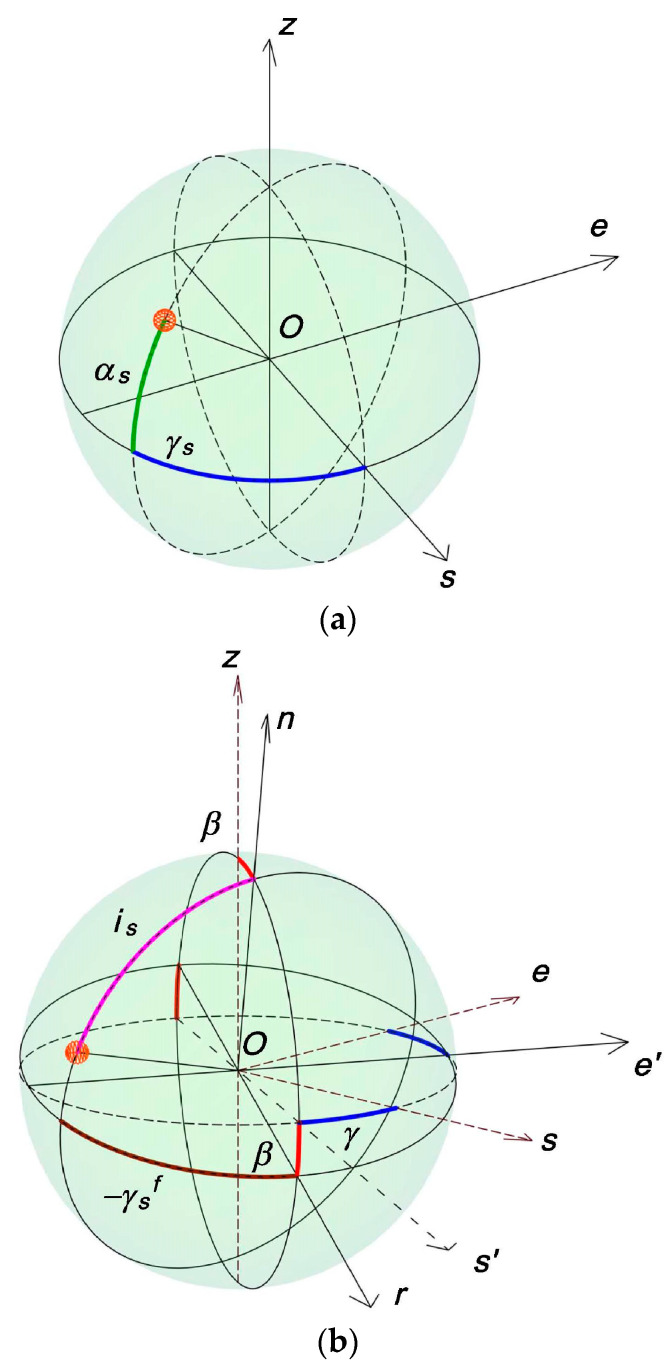
Suggestive illustration of the meaning of the angles in Equations (2)–(8). (**a**) the solar angles giving the position of the sun (on the sphere whose center is our location O on the globe) associated horizontally local plane, Osez; (**b**) Cartesian frame associated with the surface receiving solar radiation Ore’n.

As one can see from Figure 2, γ and β are, for the receiving surface, the azimuth and the inclination to the local horizontal plane (LHP), respectivley, whose Cartesian frame is Osez (where Os is the axis to the south in LHP, Oe is the axis to the east in LHP, and Oz is normal at LHP that passes through location O on the globe). α_s_ and γ_s_ are the spherical angles, called solar altitude and solar azimuth, respectively, associated with the same system of LHP, Osez. In the Cartesian frame, the Ore’n associated with the incline planar receiving surface (of azimuth γ and inclination β) Or and Oe’ are in the plane of the receiving surface, and On is normal to the exterior face of the receiving surface. Or and Oe’ are analog axes for the inclined surface of the Os and, respectively, Oe for the horizontal receiving surface. The solar spherical angles associated with this Cartesian frame are the “solar azimuth” γ_s_^f^ and the “solar altitude” (90°—i_s_).

The calculation relationships in Equations (3)–(7) were taken from [39], and the relationship for determining the incidence angle of the sun rays, Equation (8), from [40].

To find out the total solar radiation, firstly, the solar azimuth and altitude (elevation) were computed for the days and hours of interest. For this purpose, the “NOAA Solar Calculator” [41] was used. The data for Bucharest (44.5-degree Latitude and 26.13-degree Longitude) and the day and time of interest were input. The solar azimuth obtained is expressed against the north.

The angles that characterize the geometric elements of the greenhouse, in the position in which it was placed when the measurements were performed, and the surface’s inclination with regard to the horizontal plane are presented in Table 1.

Using Equation (8) and the values in Table 1, i_s_, the sun rays’ incidence angle, was determined. Care was taken to ensure that the solar azimuth angle, γ_s_, was expressed relative to the south, as considered in Equation (8). The i_s_ angle was determined for each day and time of day and for each orientation.

After that, Equations (3)–(7) were applied, and the beam, diffuse, and reflected radiations were determined for all tilted surfaces of various orientations for the days and hours to be studied.

τ, the transmissivity of the mini-greenhouse material, was included in the calculation of the total solar radiation. It depends not only on the material, but also on the incidence angle of the sun rays, i_s_. Using Window software (Version 7.8.71, 2023) [42], the transmissivity was computed for different incidence angles. These values can be seen in Table 2.

When the fraction of global total radiation, G, the global diffuse radiation, G_d_, was not known, it was found using the diffuse radiation calculation model of Erbs [43], see Equations (9)–(13). The notations used the Erbs and for all models are in accordance with the notations used in this paper. The extraterrestrial global horizontal solar irradiance, G_etr_, which is used in the Erbs calculation, was obtained for Bucharest, as well as the desired day and time with the calculation provided by NREL [44].

(9) $$k_{t}=\frac{G}{G_{etr}}$$

(10) $$k_{t}\leq 0.22;k_{d}=1-0.09\cdot k_{t}$$

(11) $$0.22\leq k_{t}\leq 0.8;\;k_{d}=0.9511-1.1604\cdot k_{t}+4.39\cdot k_{t}^{2}-16.64\cdot k_{t}^{3}+12.34\cdot k_{t}^{4}$$

(12) $$k_{t}>0.8;k_{d}=0.165$$

(13) $$k_{d}=\frac{G_{d}}{G}$$

where:

G [W/m^2^]—global irradiance on the horizontal plane received per unit area and per unit time;
G_etr_ [W/m^2^]—extraterrestrial global horizontal solar irradiance;
G_d_ [W/m^2^]—diffuse horizontal irradiance;
k_t_—clearness index;
k_d_—diffuse fraction.

The latent heat energy flow due to plant evapotranspiration, Q_e_, is calculated with the following formula:

(14) $$Q_{e}=M_{T}\cdot L_{v}\cdot A_{sol}$$

where:

M_T_ [kgH_2_O/s·m^2^]—rate of transpiration (2.8);
L_v_ [J/kgH_2_O]—latent heat of the vaporization of water (2,265,000);
A_sol_ [m^2^]—the ground area of the mini-greenhouse (0.6·0.45 = 0.27).

The transpiration rate is a complex phenomenon that depends not only on the type of culture, but also on many other factors. It is difficult to compute, and the values are mostly determined experimentally. The plant transpiration rate in the mini-greenhouse was assimilated with that presented in [16]. For this purpose, the graph of the transpiration rate according to the infusion time was used. Thus, the considered value was 2.8 kgH_2_O/s·m^2^.

The heat energy flow by conduction and convection, Q_cond_, is calculated with the following formula:

(15) $$Q_{cond}=U\cdot A_{ext}\cdot (T_{in}-T_{out})$$

where:

U [W/m^2^·K]—mini-greenhouse thermal transmittance (5.586);
A_ext_ [m^2^]—exterior area of the mini-greenhouse (1.39, calculated using the geometric model);
T_in_ [°C]—indoor air temperature (will be computed);
T_out_ [°C]—outdoor air temperature (see Table A2).

The thermal transmittance of the greenhouse envelope adjusted for the thermal bridges had to be determined. There are studies that show that, in percentage terms, the losses through thermal bridges become higher and higher as the thermal resistance of the closing surfaces increases [45].

**Figure 3:**
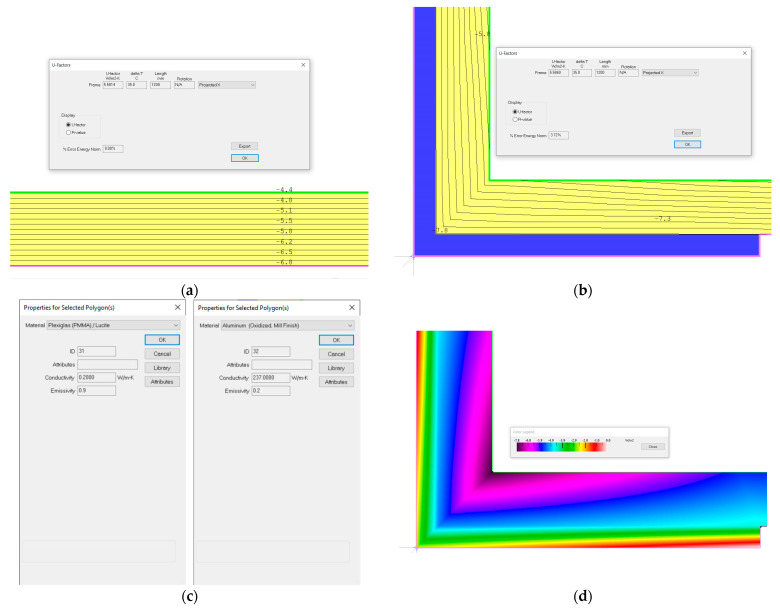
Images from the simulation in THERM. (**a**) The plexiglass wall without the thermal bridges and the transmittance calculated value; (**b**) The plexiglass wall with a corner profile, the isotherms resulting from the simulation, and the calculated transmittance value; (**c**) The materials used in the simulation (Plexiglas and aluminum) and their characteristics; (**d**) The plexiglass wall with a corner profile, with the simulated heat flow displayed as “color flow magnitude”.

Being small, the mini-greenhouse has only corner profiles as structural elements, attached with screws to the Plexiglas material from which the mini-greenhouse walls are made. A thermal simulation was carried out in the THERM Finite Element Simulator [46] software (Version 7.7.10.0, 2019). We wanted to see how much the thermal resistance of the external walls changes due to the linear thermal bridges created by the corner profiles. Details about the simulation in the THERM software can be found in Figure 3.

For the wall with the corner profile, U = 5.586 W/m^2^·K, compared to U = 5.581 W/m^2^·K for the wall without it. This increase in the thermal transmittance, U (which means a decrease in the thermal resistance R = 1/U), occurs due to the thermal bridge created by the corner profile. This increase in thermal transmittance due to the thermal bridges is not significant. In the calculations, the corrected value of U = 5.586 W/m^2^·K is used.

In general, air exchange can take place through infiltration or ventilation. Only the air exchange through infiltration was considered here, because there is no ventilation system in this study.

The heat energy flow due to air exchange, Q_v_, is calculated with the following formula:

(16) $$Q_{v}=0.33\cdot n_{a}\cdot V\cdot (T_{in}-T_{out})$$

where:

0.33 [h·J/(kg·K)]—coefficient that takes into account the seconds–hours transformation, density, and specific heat of the air;
n_a_ [h^−1^]—number of air changes per hour (0.5);
V [m^3^]—greenhouse volume (0.162, calculated using the geometric model).

The number of air changes per hour depends on the type of construction and is quite difficult to find. In demanding situations, like Passivhaus building certification, blower door tests are required [47]. In this paper, an estimated value of 0.5 h^−1^ was taken, using NP048 [48], for a “sheltered” construction, as was the mini-greenhouse during the measurements.

The heat energy flow by longwave radiation, Q_t_, is calculated with the following formula:

(17) $$Q_{t}=\sigma \cdot A_{sol}\cdot \tau \cdot ({\varepsilon_{i}\cdot T}_{in}^{4}-{\varepsilon_{cer}\cdot T}_{cer}^{4})$$

where:

σ [W/m^2^·K^4^]—Stefan–Boltzmann’s constant (5.67 × 10^−8^);
ε_i_ []—emissivity of the interior greenhouse material (0.94);
ε_cer_ []—apparent emissivity of the sky (Equation (18));
e_0_ [Pa]—outdoor vapor pressure at T_o_ (see Table A2);
T_o_ [K]—outdoor temperature at a standard height (considered equal to the outdoor temperature);
T_cer_ [K]—sky temperature (Equation (19)).

(18) $$\varepsilon_{cer}=0.70+{5.95\cdot 10}^{-7}\cdot e_{0}\cdot exp\left(\frac{1500}{T_{o}}\right)$$

(19) $$T_{cer}=0.0552{\left(T_{o}\right)}^{1.5}$$

An equation of degree 4, with the unknown T_in_, was obtained. This was solved in MsExcel, using the add-in named Solver. As a verification of the correctness of the calculations, these were also performed in Mathcad.

For the study of a greenhouse, which is sensitive to hourly variations of environmental parameters, the calculation made using monthly average values is not conclusive; therefore, using the model in this paper, the calculations were made using hourly values for the outdoor temperature (dry bulb temp (°C)), for the global horizontal irradiance on the horizontal plane [W·h/m^2^], and for the diffuse horizontal irradiance on the horizontal plane [W·h/m^2^]. The mentioned parameters were taken from an IWEC weather file [49]. The vapor saturation pressure was obtained based on temperature using the CALOREX calculator [50].

The same calculation algorithm used for determining the internal temperature of the greenhouse was also used for the climatic data from Tutiempo [51]. In this situation, only the global horizontal irradiance on the horizontal plane was available, so a calculation model was used to determine the diffuse irradiance component. The model is presented in Equations (9)–(13).

## 3. Results and Discussion

### 3.1. Results on the ThingSpeak Platform

**Figure 4:**
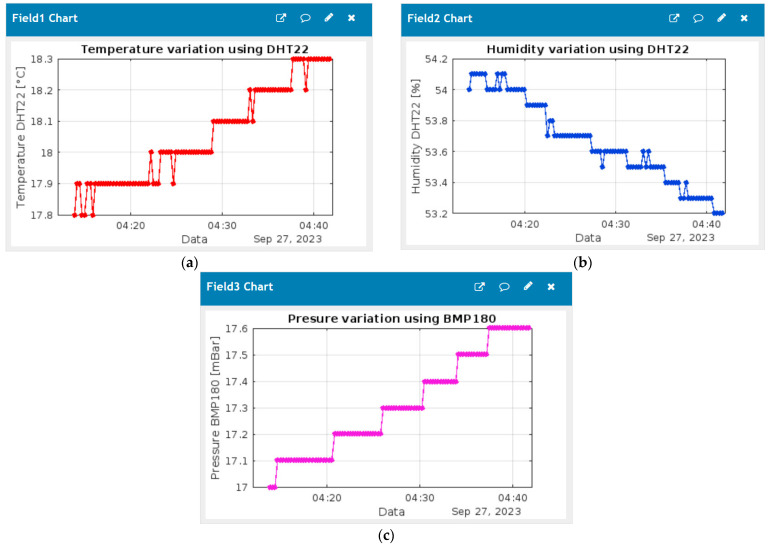
The values read by the sensors, as seen in the ThingSpeak platform: (**a**) Temperature (°C); (**b**) Humidity (%); (**c**) Pressure (mBar).

The data transmitted to the ThingSpeak application, displayed in the form of graphs (a facility offered by this platform), can be seen in Figure 4.

The data presented in the form of a dashboard, Figure 4, are very suggestive. This is useful if we want to have an overview and follow the data in real time, as well as their evolution over time. In this paper, the numerical values are of more interest than the graphs. The sensor system developed in [37] contains other fields (read or computed) that are not of interest for this study and, for that reason, are not presented here. For a better visualization of the results in graphic form, the possibilities resulting from the integration of ThingSpeak [32] with MatLab [51] were used. The graphics automatically generated by ThingSpeak were customized in MatLab. The changes made refer to the chart area and the way the values are displayed on the y axis.

A selection from the data recorded by the sensors, for the days of 12 July 2022, 3 and 4 July 2023, 16 and 17 August 2023, and 26 and 27 September 2023 are shown in Table A1. The relevant measured data were selected at about half an hour. The corresponding resulting values that were obtained by solving the energy balance equation are shown too. The sampling time of 15 s for the JSON records is far too small.

### 3.2. Comparison of Calculations with Measurements

The internal temperature values obtained after applying the energy balance, in both cases, using the IWEC weather file or Tutiempo, can be seen in Appendix A, Table A2 and Table A3.

**Figure 5:**
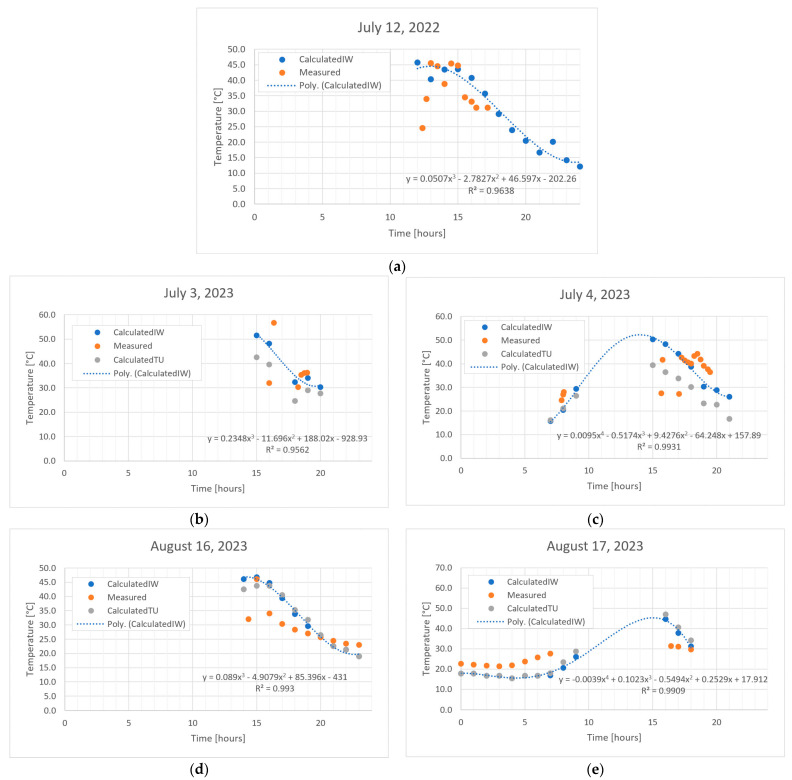

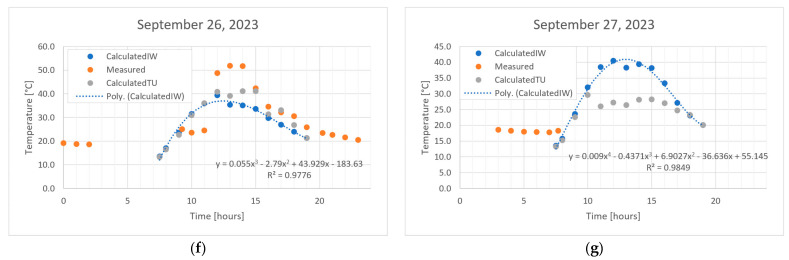
Comparison of calculations with measurements: (**a**) 12 July 2022; (**b**) 3 July 2023; (**c**) 4 July 2023; (**d**) 16 August 2023; (**e**) 17 August 2023; (**f**) 26 September 2023; (**g**) 27 September 2023.

The graphs in Figure 5 represent the comparison between the measured and calculated values. As can be seen from the legend, for all three of the graphs, there are curves made of points, named “CalculatedIW.” They represent the temperature calculated using the greenhouse energy balance and environmental parameters from IWEC, for which the data from Table A2 are used. In addition, for all of the graphs, there are also curves made of points named “Measured”, which represent the measured values. Except for the first graph, there are also the “CalculatedTU” curves made of points that represent the temperature calculated using the greenhouse energy balance and the environmental parameters from Tutiempo [52], for which the data from Table A3 were used. The graphs are of the scatter (x, y) type, where the time at which the measurement took place is represented on the abscissa axis, and the temperature is expressed in Celsius degrees on the ordinate axis. The hours were transformed from minutes and seconds into the decimal format. For a correct representation, see the last column of Table A1.

For 12 July 2022, at the time interval of 13–15, there is a good match between the data recorded by the sensors and the data computed using the greenhouse energy balance, as can be seen in Figure 5. The sky was cloudy after 3 pm, when the measurements were taken. Knowing that the IWEC file is made up of weather data from certain characteristic years, in order to avoid small discrepancies between IWEC and the external parameters at the time of the measurements being taken, in future studies, a pyranometer [53] could be used to directly measure the solar radiation. Otherwise, a model can also be used to calculate solar radiation, depending on the location and day/time of year [54]. Knowing that the IWEC file is made up of weather data from certain characteristic years, in order to avoid small discrepancies between IWEC and external parameters at the time of measurements, in future studies a pyranometer [53], to directly measure solar radiation, can be used. On-site measurements are better than data measured by the nearest weather station.

For 3 and 4 July, 16 and 17 August, and 26 and 27 September 2023, in addition to the IWEC file, the real data for Bucharest/Imh, taken from Tutiempo [52], were also obtained, as can be seen in Figure 5. In Appendix A, Table A3, one can see the indoor temperature values obtained following the application of energy balance, in the case of using the environmental parameters obtained from Tutiempo.

Measurements were also taken for shorter periods of a few hours, as well as measurements for a day, namely 24 h. A good fit of the data can be observed, and the values that are far from the “main trend”, we can call them outliers, could be caused by the fact that it takes a while for the system to reach equilibrium. It is possible that those measurements were made too early and that not enough time had passed since the beginning of the measurements.

In order to compare the obtained results with similar ones in the specialized literature, similar articles were searched. For example, in [55], the greenhouse calculations were performed using a dynamic physical simulation model with a single- and double-layer greenhouse under Dutch weather conditions. The comparison was made based on the energy consumption and the costs. Ref. [56] deals with the design and performance assessment of solar greenhouses for mushroom ventilation, and this study developed a 3D mathematical model suitable for a large-scale park of mushroom solar greenhouses based on computational fluid dynamics (CFD) theory. A computational greenhouse model that was developed using inputs from the real design, materials, and location of a Purdue Lily greenhouse in West Lafayette, Indiana, is presented in [57]. Here, the emphasis falls on the optimal location of the plant pots in the greenhouse. In [58], a two-dimensional steady-state simulation is carried out but, unlike in our case, in a strongly ventilated greenhouse, with a wind speed exceeding 1 m·s^−1^. The model was verified by comparing the numerical results with the experimental data. Although the models developed in these works are complex, using CFD simulation, they refer to large greenhouses. To our knowledge, simple mathematical models for the energy balance of a small greenhouse (mini-greenhouse) have not yet been tried.

## 4. Conclusions

In this paper, a simple energy balance model and a set of deployed “mini-greenhouses”, motorized in real time, are used as tools for making decisions in the early faze design of a real-size greenhouse. The presented tools are used to find out the position and orientation of the real-size greenhouse. The novelty exists in testing the optimal position from the point of view of the internal temperature both through a mathematical model and through the creation of mini-greenhouses that have low cost requirements. Among the locations and orientations proposed for the real-size greenhouse, some were eliminated by running the mathematical model. After that, the mini-greenhouses could be placed in the rest of the locations and, following the measurements made with them, the position for the real-scale greenhouse could be chosen. For small greenhouse measurements, a centralized data transmission system is proposed, which helps in monitoring and decision making. The proposed mathematical model is a simple one, and it is easy to apply considering that it only contributes to the elimination of the most unfavorable positions. Energy conservation for a greenhouse was applied by identifying the terms that go into the balance equation and, thus, determining the indoor temperature in the greenhouse. The results obtained after the steady-state simulation of a greenhouse are compared with the values obtained from the measurements of the indoor climate parameters in a greenhouse. Although we can say that the mathematical model is validated, the period for which that was carried out was quite short, and it is recommended to perform measurements over longer periods of time and at other times of the year. Depending on the results obtained, it is possible to carry out, if necessary, the calibration of the mathematical model. In the future, we proposed to mount the sensor system in a full-size greenhouse and compare its behavior to that of the mini-greenhouse.

## Figures and Tables

**Table 1:** The geometric elements of the greenhouse (entry data in calculations).

		Area [m^2^]	β [Degree]	γ [Degree]	β [Rad]	γ [Rad]
North wall, nw	nw	0.30	90	180.00	1.57	3.14
North roof, nr	nr	0.15	24	180.00	0.42	3.14
East wall, ew	ew	0.25	90	90.00	1.57	1.57
South wall, sw	sw	0.30	90	0.00	1.57	0.00
South roof, sr	sr	0.15	24	0.00	0.42	0.00
West wall, ww	ww	0.25	90	270.00	1.57	4.71

**Table 2:** Optical properties for a glazing system composed of Plexiglas (acrylic), 3 mm.

Angle	Tvis:	Rfvis:	Rbvis:	τ	Rfsol:	Rbsol:	Abs1:	SHGCc:
0	0.923	0.074	0.074	**0.849**	0.069	0.069	0.082	0.875
10	0.923	0.074	0.074	**0.849**	0.069	0.069	0.082	0.874
20	0.922	0.074	0.074	**0.848**	0.069	0.069	0.083	0.874
30	0.92	0.076	0.076	**0.845**	0.071	0.071	0.084	0.871
40	0.914	0.082	0.082	**0.838**	0.077	0.077	0.085	0.864
50	0.895	0.1	0.1	**0.819**	0.095	0.095	0.086	0.846
60	0.847	0.148	0.148	**0.773**	0.14	0.14	0.086	0.8
70	0.728	0.268	0.268	**0.663**	0.255	0.255	0.082	0.688
80	0.457	0.538	0.538	**0.414**	0.519	0.519	0.068	0.435
90	0	1	1	**0**	1	1	0	0
Hemis	0.846	0.14	0.14	**0.774**	0.133	0.133	0.083	0.8

Transmissivity is highlighted in bold.

## Data Availability

Data are contained within the article.

## Appendix A

**Table A1:** Data recorded by the sensors—selection.

“created_at:”	“entry_id:”	“field1:” (Temperature, °C)	“field2:” (Humidity, %)	“field4:” (Pressure, mBar)	Local Time (Bucharest)	Time (Decimal)
2022-07-12T09:23:52Z	84158	24.6	41.7	1012.69	12:23	12.38
2022-07-12T09:40:43Z	84169	34	36	1007.87	12:40	12.67
2022-07-12T10:00:15Z	84236	45.5	23.9	1007.66	13:00	13
2022-07-12T10:30:06Z	84338	44.5	23.8	1009.13	13:30	13.5
2022-07-12T11:00:01Z	84441	38.8	26.4	1009.32	14:00	14
2022-07-12T11:30:15Z	84545	45.4	22.6	1009.68	14:30	14.5
2022-07-12T12:00:04Z	84646	44.8	23.2	1010.54	15:00	15
2022-07-12T12:30:07Z	84749	34.5	26.3	1011.53	15:30	15.5
2022-07-12T13:00:07Z	84850	33.1	26	1011.36	16:00	16
2022-07-12T13:21:41Z	84924	31.1	27.8	1011.16	16:21	16.35
2022-07-12T14:11:38Z	84925	31.2	27.8	1011.09	17:11	17.18
2023-07-03T13:00:09Z	85452	32	49.5	997.4	16:00	16
2023-07-03T13:22:24Z	85534	56.7	25.9	993.9	16:22	16.37
2023-07-03T15:15:00Z	85585	30.3	52.4	1000.1	18:15	18.25
2023-07-03T15:30:23Z	85641	35.3	44.5	1000.44	18:30	18.5
2023-07-03T15:45:00Z	85695	36.1	44	1000.69	18:45	18.75
2023-07-03T15:57:39Z	85741	36.3	43.8	1000.54	18:57	18.95
2023-07-04T04:52:52Z	85743	24.6	66.9	1004.09	7:52	7.87
2023-07-04T05:00:00Z	85763	27	61.8	1003.97	8:00	8
2023-07-04T05:03:17Z	85775	28.1	58.6	1004.07	8:03	8.05
2023-07-04T12:40:06Z	85787	27.5	58	998.59	15:40	15.67
2023-07-04T12:47:41Z	85814	41.7	36.1	998.84	15:47	15.78
2023-07-04T14:03:54Z	85825	27.2	57.4	1001.4	17:03	17.05
2023-07-04T14:15:06Z	85858	42.7	35	1002.02	17:15	17.25
2023-07-04T14:30:08Z	85913	41.4	35.3	1002.14	17:30	17.5
2023-07-04T14:45:12Z	85968	40.6	35.1	1002.03	17:45	17.75
2023-07-04T15:00:14Z	86023	40.1	36.4	1001.86	18:00	18
2023-07-04T15:15:04Z	86050	43.3	33.6	1001.37	18:15	18.25
2023-07-04T15:30:00Z	86084	44.2	33.9	1001.49	18:30	18.5
2023-07-04T15:45:13Z	86140	41.8	34.4	1001.56	18:45	18.75
2023-07-04T16:00:10Z	86195	39.1	36.4	1001.76	19:00	19
2023-07-04T16:15:07Z	86250	37.7	38.1	1001.7	19:18	19.3
2023-07-04T16:29:47Z	86304	36.5	39.8	1001.82	19:29	19.48
2023-08-16T14:22:39Z	90350	32	49.7	1004.9	17:22	14.37
2023-08-16T15:00:06Z	90483	46.1	28	1005.52	18:00	15
2023-08-16T16:00:12Z	90698	34	37	1006.08	19:00	16
2023-08-16T17:00:01Z	90909	30.4	41.3	1006.43	20:00	17
2023-08-16T18:00:24Z	91114	28.4	44.2	1006.59	21:00	18
2023-08-16T19:00:03Z	91322	27	47.7	1006.79	22:00	19
2023-08-16T20:00:07Z	91532	25.7	52.4	1006.9	23:00	20
2023-08-16T21:00:13Z	91744	24.5	55.2	1006.59	0:00	21
2023-08-16T22:00:06Z	91958	23.4	58.8	1006.5	1:00	22
2023-08-16T23:00:04Z	92160	23	62	1006.27	2:00	23
2023-08-17T00:00:11Z	92375	22.7	63.6	1006.18	3:00	0
2023-08-17T01:00:07Z	92590	22.1	65.8	1005.83	4:00	1
2023-08-17T02:00:12Z	92806	21.7	66.5	1005.89	5:00	2
2023-08-17T03:00:06Z	93019	21.4	68.6	1005.88	6:00	3
2023-08-17T04:00:11Z	93232	21.9	68.2	1005.96	7:00	4
2023-08-17T05:00:09Z	93445	23.8	63.6	1006.23	8:00	5
2023-08-17T06:00:00Z	93658	25.7	59.7	1006.02	9:00	6
2023-08-17T07:00:03Z	93874	27.6	56.6	1006.34	10:00	7
2023-08-17T08:31:05Z	94009	47.7	28	1006.9	11:00	8
2023-08-17T09:00:08Z	94111	57.3	24.2	1004.2	12:00	9
2023-08-17T16:26:56Z	94130	31.4	37.6	1005.07	19:26	16.43
2023-08-17T17:00:07Z	94247	31.1	38.5	1005.14	20:00	17
2023-08-17T18:00:07Z	94461	29.7	40.7	1005.51	21:00	18
2023-09-26T06:16:07Z	95753	25	42.1	1011.22	9:16	9.27
2023-09-26T07:00:12Z	95858	23.6	44.8	1011.19	10:00	10
2023-09-26T08:00:12Z	96018	24.5	43.1	1011.16	11:00	11
2023-09-26T09:46:25Z	96036	48.8	23.4	1010.4	12:00	12
2023-09-26T10:00:14Z	96086	51.8	22.6	1010.7	13:00	13
2023-09-26T11:00:08Z	96301	51.7	21.6	1010.62	14:00	14
2023-09-26T12:00:12Z	96516	42.3	23.8	1010.03	15:00	15
2023-09-26T13:00:10Z	96731	34.6	27	1010.33	16:00	16
2023-09-26T14:00:09Z	96945	32.2	27.1	1010.45	17:00	17
2023-09-26T15:00:18Z	97160	30.6	29.3	1010.36	18:00	18
2023-09-26T16:00:03Z	97371	25.8	34.2	1010.52	19:00	19
2023-09-26T17:14:05Z	97400	23.5	40.5	1011.15	20:14	20.23
2023-09-26T18:00:14Z	97563	22.7	42.7	1011.39	21:00	21
2023-09-26T19:00:09Z	97777	21.6	45.8	1011.42	22:00	22
2023-09-26T20:00:01Z	97990	20.5	48.6	1011.53	23:00	23
2023-09-26T21:00:01Z	98202	19.2	51.8	1011.52	0:00	0
2023-09-26T22:00:10Z	98431	18.8	54.2	1011.25	1:00	1
2023-09-26T23:00:09Z	98627	18.6	54.7	1011.03	2:00	2
2023-09-27T00:00:01Z	98835	18.6	54	1010.76	3:00	3
2023-09-27T01:00:05Z	99040	18.3	53.7	1010.46	4:00	4
2023-09-27T02:00:11Z	99248	18	52.9	1010.2	5:00	5
2023-09-27T03:00:01Z	99462	17.9	53.5	1010.18	6:00	6
2023-09-27T04:00:02Z	99673	17.8	54.2	1010.23	7:00	7
2023-09-27T04:41:40Z	99822	18.3	53.2	1010.42	7:41	7.68

**Table A2:** Data computed using the greenhouse energy balance and environmental parameters from IWEC.

Date	Hour	Tin [°C]	Tin [K]	Tout [°C]	Global Horizontal Radiation [Wh/m^2^]	Water Vapor Saturation Pressure [Pa]
12 July 2022	12	45.77	318.92	28	205.22	3781
	13	40.30	313.45	26	175.50	3362
	14	43.50	316.65	27.9	184.92	3759
	15	43.54	316.69	28.2	181.93	3826
	16	40.71	313.86	29	146.43	4007
	17	35.65	308.80	28.6	103.53	3916
	18	29.08	302.23	27.3	57.00	3630
	19	23.91	297.06	26	23.65	3362
	20	20.48	293.63	25	3.06	3167
	21	16.68	289.83	22	0.00	2664
	22	20.14	293.29	25	0.00	3167
	23	14.16	287.31	19.8	0.00	2310
	24	12.15	285.30	18	0.00	2064
3 July 2023	15	51.52	324.67	35	179.14	5627
	16	48.09	321.24	34.9	147.59	5596
	18	32.36	305.51	34.9	0.00	5596
	19	34.04	307.19	34	26.41	5322
	20	30.33	303.48	33	3.87	5033
4 July 2023	7	15.78	288.93	19	22.70	2197
	8	20.46	293.61	19.4	61.13	2253
	9	29.34	302.49	23	105.43	2810
	15	50.28	323.43	34	179.30	5323
	16	48.19	321.34	35	147.39	5627
	17	44.26	317.41	35.1	108.87	5658
	18	38.81	311.96	35	58.84	5627
	19	30.35	303.50	33	4.07	5033
	20	28.82	301.97	31.8	3.87	4704
	21	26.03	299.18	29.9	0.00	4221
5 July 2023	17	25.83	298.98	21.6	87.70	2580
	18	21.25	294.40	21	51.95	2487
	19	18.25	291.40	21	24.71	2487
16 August 2023	14	46.09	319.24	31	174.48	4495
	15	46.77	319.92	32	169.43	4758
	16	44.79	317.94	33	139.00	5033
	17	39.45	312.60	33	88.73	5033
	18	33.81	306.96	33	36.07	5033
	19	29.64	302.79	32	9.13	4758
	20	26.19	299.34	30	0.37	4245
	21	22.51	295.66	27	0.00	3567
	22	21.32	294.47	26	0.00	3362
	23	18.97	292.12	24	0.00	2984
17 August 2023	0	17.81	290.96	23	0.00	2810
	1	17.81	290.96	23	0.00	2810
	2	16.66	289.81	22	0.00	2644
	3	16.66	289.81	22	0.00	2644
	4	15.52	288.67	21	0.00	2487
	5	16.66	289.81	22	0.00	2644
	6	16.66	289.81	22	0.00	2644
	7	16.78	289.93	22	1.10	2644
	8	20.55	293.70	24	14.36	2984
	9	26.03	299.18	26	43.10	3362
	16	44.72	317.87	33	138.33	5033
	17	37.81	310.96	32	85.01	4758
	18	31.21	304.36	31	35.21	4495
26 September 2023	7.5	13.48	286.63	19	1.90	2197
	8	16.96	290.11	20	23.22	2338
	9	23.53	296.68	21	72.81	2487
	10	31.52	304.67	24	115.04	2984
	11	35.96	309.11	25	145.61	3169
	12	39.38	312.53	28	145.03	3781
	13	35.43	308.58	28	108.15	3781
	14	35.10	308.25	29	93.97	4007
	15	33.57	306.72	29	79.76	4007
	16	29.79	302.94	29	44.76	4007
	17	26.93	300.08	29	18.39	4007
	18	23.98	297.13	28	2.48	3781
	19	21.32	294.47	26	0.00	3362
27 September 2023	7.5	13.53	286.68	19	2.35	2197
	8	15.77	288.92	19	22.59	2197
	9	23.56	296.71	21	73.11	2487
	10	32.07	305.22	24	120.12	2984
	11	38.49	311.64	26	158.51	3362
	12	40.53	313.68	27	166.76	3567
	13	38.32	311.47	27	146.11	3567
	14	39.40	312.55	28	145.18	3781
	15	38.22	311.37	28	134.15	3781
	16	33.37	306.52	28	88.98	3781
	17	27.11	300.26	27	42.16	3567
	18	23.11	296.26	27	5.49	3567
	19	20.14	293.29	25	0.00	3169

**Table A3:** Data computed using the greenhouse energy balance and environmental parameters from Tutiempo.

Date	Hour	Tin [°C]	Tin [K]	Tout [°C]	Global Horizontal Radiation [Wh/m^2^]	Water Vapor Saturation Pressure [Pa]
3 July 2023	15	42.53	315.68	31	140.92	4495
	16	39.58	312.73	30	124.54	4245
	18	24.60	297.75	28	0.00	4758
	19	28.97	302.12	29	37.11	4007
	20	27.72	300.87	29	25.68	4007
4 July 2023	7	16.15	289.30	18	36.06	2064
	8	21.05	294.20	19	70.49	2197
	9	26.38	299.53	21	98.95	2487
	15	39.47	312.62	27	156.82	3567
	16	36.49	309.64	27	128.97	3567
	17	33.86	307.01	27	104.50	3567
	18	30.15	303.30	27	70.17	3567
	19	23.28	296.43	27	7.00	3567
	20	22.67	295.82	25	23.03	3168
	21	16.66	289.81	22	0.00	2644
5 July 2023	17	32.96	306.11	34	38.99	5323
	18	35.08	308.23	35	24.04	5627
	19	31.61	304.76	33	15.69	5033
16 August 2023	14	42.56	315.71	31	141.20	4495
	15	43.80	316.95	32	141.31	4758
	16	43.80	316.95	33	139.29	5033
	17	40.52	313.67	33	98.71	5033
	18	35.26	308.41	33	49.57	5033
	19	31.76	304.91	32	28.80	4758
	20	26.45	299.60	30	2.74	4245
	21	22.51	295.66	27	0.00	3567
	22	21.32	294.47	26	0.00	3362
	23	18.97	292.12	24	0.00	2984
17 August 2023	0	17.81	290.96	23	0.00	2810
	1	17.81	290.96	23	0.00	2810
	2	16.66	289.81	22	0.00	2644
	3	16.66	289.81	22	0.00	2644
	4	15.52	288.67	21	0.00	2487
	5	16.66	289.81	22	0.00	2644
	6	16.66	289.81	22	0.00	2644
	7	17.96	291.11	22	11.73	2644
	8	23.48	296.63	24	41.09	2984
	9	28.67	301.82	26	67.34	3362
	16	46.94	320.09	33	159.38	5033
	17	40.65	313.80	32	111.69	4758
	18	34.23	307.38	31	63.21	4495
26 September 2023	7.5	13.26	286.41	19	0.00	2197
	8	16.49	289.64	20	18.98	2338
	9	22.71	295.86	21	65.28	2487
	10	30.92	304.07	24	109.49	2984
	11	36.04	309.19	25	146.42	3168
	12	40.84	313.99	28	158.73	3781
	13	39.18	312.33	28	143.20	3781
	14	41.14	314.29	29	150.43	4007
	15	41.15	314.30	29	150.51	4007
	16	31.29	304.44	29	58.60	4007
	17	33.03	306.18	29	74.67	4007
	18	26.78	299.93	28	28.08	3781
	19	21.32	294.47	26	0.00	3362
27 September 2023	7.5	13.26	286.41	19	0.00	2197
	8	15.32	288.47	19	18.52	2197
	9	22.60	295.75	21	64.28	2487
	10	29.64	302.79	24	97.72	2984
	11	26.07	299.22	26	43.46	3362
	12	27.25	300.40	27	43.43	3567
	13	26.47	299.62	27	36.25	3567
	14	28.14	301.29	28	40.63	3781
	15	28.19	301.34	28	41.08	3781
	16	27.05	300.20	28	30.61	3781
	17	24.71	297.86	27	20.09	3567
	18	23.27	296.42	27	6.90	3567
	19	20.14	293.29	25	0.00	3168
